# Association between shock index and postpartum hemoglobin level and blood transfusion requirement in women delivering at public hospitals in Shiraz, 2023–2024

**DOI:** 10.1186/s12884-026-09323-7

**Published:** 2026-05-29

**Authors:** Maryam Mirzaabdollahi, Fatemeh Rahmanian, Roksana Janghorban, Parvin GhaemMaghami

**Affiliations:** 1 Master of Midwifery, Faculty of Nursing and Midwifery, Shiraz University of Medical Sciences, Shiraz, Iran; 2 Department of Midwifery and Reproductive Health, School of Nursing and Midwifery, Shiraz University of Medical Sciences, Shiraz, Iran; 3 Department of Midwifery, School of Nursing and Midwifery, Maternal-Fetal Medicine Research Center, Shiraz University of Medical Sciences, Shiraz, Iran; 4 Biostatistics PhD, School of Nursing and Midwifery, Shiraz University Medical Sciences, Shiraz, Iran

**Keywords:** Postpartum hemorrhage, SI, Maternal mortality, Hemoglobin, Blood transfusion, Delivery

## Abstract

**Background:**

Postpartum hemorrhage is a leading cause of maternal morbidity and mortality worldwide, affecting over 14 million women annually and causing approximately 80,000 preventable deaths.The Shock Index (SI), calculated as the ratio of heart rate to systolic blood pressure, is a simple, non-invasive tool for detecting early hemodynamic instability. Despite its potential clinical usefulness, few studies have examined the association between SI, postpartum hemoglobin levels, and blood transfusion requirements. This study evaluated the association between SI and these outcomes among women delivering in public hospitals in Shiraz, Iran, during 2023–2024.

**Methods:**

This retrospective cross-sectional study analyzed data from 370 women who delivered between 2023 and 2024 at three tertiary hospitals in Shiraz. Inclusion criteria included Iranian nationality, age > 18 years, delivery at or beyond 22 weeks’ gestation (regardless of neonatal status), and no comorbidities(e.g., pre-eclampsia, gestational hypertension, diabetes, or thyroid disorders). The SI was calculated at the fourth 15-minute interval post-delivery and categorized as low (< 0.70), moderate (0.70–0.89), or high (≥ 0.90). Demographic and clinical data, pre- and postpartum hemoglobin levels, and transfusion status were extracted from medical records. Analyses were performed using chi-square and two-way ANOVA tests in SPSS version 22.

**Results:**

Among the 370 women included in the study, 42 required blood transfusion. In analyses stratified by delivery type (vaginal with or without episiotomy and cesarean section), categorized SI was not significantly associated with postpartum hemoglobin decline or transfusion requirement (*P* > 0.05). However, in simple group comparisons, women who required transfusion had significantly higher mean heart rates (88.1 ± 17.8 vs. 81.9 ± 12.4 beats/min), lower systolic blood pressure (108.6 ± 7.1 vs. 114.8 ± 8.1 mmHg), and higher SI values (0.81 ± 0.18 vs. 0.72 ± 0.11) compared with the non‑transfusion group (*P* < 0.001).

**Conclusion:**

This study found that women who required blood transfusion had higher heart rates, lower systolic blood pressure, and higher SI than those who did not. However, no significant associations were observed between categorized SI and postpartum hemoglobin levels or transfusion requirement within delivery-type subgroups. Because this study evaluated associations only and did not assess diagnostic accuracy, the SI cannot be considered a screening tool based on these findings. Further prospective studies with larger sample sizes and standardized data collection are needed to evaluate the diagnostic performance of the SI and clarify its potential role in the early identification of postpartum hemorrhage.

**Supplementary Information:**

The online version contains supplementary material available at 10.1186/s12884-026-09323-7.

## Introduction

Postpartum hemorrhage continues to cause significant maternal morbidity and mortality worldwide, even with advancements in obstetric care [1]. In Iran, the maternal mortality ratio due to PPH ranges from 15.8 to 384 per 100,000 live births, reflecting regional disparities in access to healthcare [2]. Globally, postpartum hemorrhage impacts over 14 million women each year, leading to approximately 80,000 deaths, 90% of which the World Health Organization deems preventable [2–5]. Most Postpartum hemorrhage -related deaths occur within the first 24 h post-delivery, emphasizing the critical need for early detection and intervention to prevent life-threatening complications [6].The Shock Index (SI), calculated as the ratio of heart rate to systolic blood pressure, is a simple, non-invasive tool for assessing circulatory compromise, requiring no specialized equipment [7]. Unlike traditional vital signs, which may remain normal despite significant blood loss in pregnancy [8]. SI offers greater sensitivity in detecting early hemodynamic instability [4]. Retrospective studies have established SI as a reliable predictor of hypotension, blood transfusion needs, and adverse outcomes, such as prolonged hospital stays and mortality [7].During pregnancy and the immediate postpartum period, physiological changes often delay the recognition of severe hemorrhage, making SI a promising early warning tool for Postpartum hemorrhage [9, 10] .Despite the potential of SI in early Postpartum hemorrhage detection, few studies have explored its predictive accuracy for postpartum hemoglobin levels and blood transfusion requirements, particularly in low-resource settings [11–14]. This study aimed to evaluate the association between SI, postpartum hemoglobin levels, and blood transfusion requirement among women delivering at public hospitals in Shiraz, Iran.

## Methods

### Study design and setting

This retrospective cross‑sectional study reviewed the medical records of 370 postpartum women between 2023 and 2024 at three public tertiary hospitals affiliated with Shiraz University of Medical Sciences (Shooshtari, Hazrat Zeynab, and Hafez hospitals). These hospitals serve as major delivery centers in southern Iran, each with specialized obstetrics and gynecology departments and high patient volumes, ensuring a diverse study population.

### Sample Size

The final sample size of 370 participants was determined based on a power analysis (α = 0.05, power = 90%) using an estimated effect size (f² = 0.10) derived from a comparable study [15], which reported an R² of 0.092 across multiple independent variables.

### Participants and eligibility criteria

The study population comprised all women who met the following inclusion criteria: Iranian nationality (due to population homogeneity); age > 18 years (to eliminate potential risk factors that could affect SI values or physiological responses (; delivery by vaginal birth (with or without episiotomy) or cesarean section; absence of medical conditions such as pre-eclampsia, pregnancy‑induced hypertension, diabetes, or thyroid disease; delivery at or beyond 22 weeks’ gestation (regardless of neonatal status); and delivery with or without instrumental assistance (forceps or vacuum). Patients with incomplete medical records were excluded.

### Data collection and variables

Data were collected daily by reviewing hospital medical records, specifically the documentation of birth records registered in the selected hospitals, using a standardized delivery checklist. Extracted data included demographic characteristics, obstetric and labor history, as well as postpartum information recorded in the medical files. A total of 834 medical records were reviewed, of which 464 were excluded due to incomplete information. Ultimately, 370 records were included in the final analysis.

Collected variables included maternal age, height, weight, vital signs (systolic and diastolic blood pressure, pulse rate, respiratory rate, and body temperature recorded at the fourth 15-minute interval after delivery), episiotomy status (presence or absence) and degree of episiotomy, neonatal birth weight,, hemoglobin before(at hospital admission) and 6 h after delivery, parity, gravidity, abortion history, gestational age at delivery, and mode of delivery. Postpartum clinical variables included uterine status (uterine condition after delivery, recorded as well-contracted, uterine atony, or active bleeding), perineal status (classified by the presence and degree of episiotomy and/or laceration), suture site (based on episiotomy and/or laceration severity), neonatal status (The condition of the newborn immediately after delivery, categorized as normal (no admission to the neonatal intensive care unit (NICU) or abnormal (admission to the NICU for any reason).), estimated blood loss, administered medications, blood products, urine output, anesthesia type, transfusion within the first hour after delivery, and use of instrumental assistance during delivery.

### Statistical analysis

Data collected from the standardized checklist were analyzed using SPSS version 22.0. Quantitative variables were described using means (standard deviations), and qualitative variables were reported as frequencies (percentages). To examine the relationship between the target variable (quantitative) and demographic characteristics, independent two-sample t-tests were used to compare means between two-level variables, and analysis of variance (ANOVA) with post-hoc pairwise comparisons was applied for multi-level variables. For assessing correlations between quantitative variables, Pearson’s correlation test was used, with Spearman’s rank correlation applied in cases of non-normality. Associations between qualitative variables were evaluated using the chi-square test.Two-way analysis of variance (Two-Way ANOVA) was used to assess the effects of categorized SI ((heart rate/systolic blood pressure) stratified into three clinically relevant categories: low < 0.70, moderate 0.70–0.89, and high ≥ 0.90), episiotomy status, and their interaction on hemoglobin levels in vaginal deliveries, while one-way analysis of variance (One-Way ANOVA) was used to examine the effect of categorized SI on hemoglobin levels in cesarean deliveries. In these models, the intercept was defined as the estimated mean value of the dependent variable (hemoglobin level) when all categorical predictors (SI category and episiotomy status) were at their reference levels. Blood loss, uterine status, perineal status, suture location, anesthesia type, blood product type, and urinary output were not analyzed due to homogeneity. A significance level of *P* < 0.05 was considered statistically significant for all analyses. 

## Results

In this retrospective cross‑sectional study, the medical records of 370 patients were reviewed. Of these, 42 patients (11.4%) received blood transfusions, while 328 patients (88.6%) did not. The qualitative demographic characteristics of patients are presented in Table 1. Based on the results of the chi-square test, no significant association was observed between episiotomy status, episiotomy degree, instrumental delivery, neonatal status, or history of abortion and receiving blood transfusion (*P* > 0.05). However, a significant association was found between parity (*P* = 0.017), mode of delivery (*P* < 0.001), and medications used (*P* < 0.001) with receiving blood transfusion.

**Table 1:** Comparison of frequency distributions of qualitative demographic variables between transfusion and non transfusion groups

Variable	Category	Total *N*(%)	Transfusion(+) *N*(%)	Transfusion(-) *N*(%)	*P*-Value
Mode of delivery	Vaginal	65(17.6)	20(47.6)	45(13.7)	< 0.0001
	Cesarean	305(82.4)	22(52.4)	267(81.4)	
Episiotomy	YES	43(66.2)	10(50.0)	33(73.3)	0.067
	NO	22(33.8)	10(50.0)	12(26.7)	
Episiotomy grade	Grade 1	40(93.0)	10(100)	30(90.9)	0.323
	Grade2	3(7)	0(0.0)	3(9.1)	
Instrumental delivery	YES	3(4.6)	2(10.0)	1(2.2)	0.222
	NO	62(95.4)	18(9.0)	44(97.8)	
Neonatal Status	no admission to the NICU	300(81.1)	33(78.6)	267(81.4)	0.067
	admission to the NICU	70(18.9)	9(21.4)	61(18.6)	
Medications	Oxytocin ONLY	359(97)	33(78.6)	326(99.4)	< 0.0001
	Oxytocin + Methylergometrine	4(1.1)	4(9.5)	0(0)	
	Oxytocin + Methylergometrine + Misoprostol	7(1.9)	5(11.9)	2(0.6)	
Abortion	0	278(75.1)	31(73.8)	247(75.3)	0.107
	1	68(18.4)	5(11.9)	63(19.2)	
	2	18(4.9)	5(11.9)	13(4)	
	3	6(1.6)	1(2.44)	5(1.5)	
Parity	0	91(24.6)	17(40.5)	74(22.6)	0.017
	1	189(51.1)	13(31)	176(53.7)	
	2	74(20)	8(19)	66(20.1)	
	3	14(3.8)	4(9.5)	10(3)	
	4	2(0.5)	0(0)	2(0.6)	

Data are presented as number (percentage). The chi-squared test was used to compare the distribution of variables between groups

A p-value of < 0.05 was considered statistically significant

Quantitative demographic characteristics are summarized in Table 2. No statistically significant differences were found between the two groups in relation to maternal age, maternal height, neonatal birth weight, gravidity, diastolic blood pressure, or gestational age (*P* > 0.05). Conversely, mean maternal weight, body temperature, systolic blood pressure, and both pre‑ and post‑partum hemoglobin levels were significantly higher in the non‑transfusion group compared with the transfusion group (*P* < 0.05). Additionally, the mean heart rate and respiratory rate were significantly higher in the transfusion group (*P* < 0.05).

**Table 2:** Comparison of mean standard deviation of quantitative demographic and clinical variables between transfusion and non transfusion groups

Variable	Total (*N* = 370)	Transfusion(+) *N* = 42	Transfusion(-) *N* = 328	*P*-Value
Age (years)	29.59 ± 6.66	28.52 ± 7.575	30.66 ± 5.762	0.084
Height (cm)	160.68 ± 5.25	161.63 ± 5.074	160.00 ± 5.439	0.127
Weight (kg)	70.30 ± 9.44	68.52 ± 7.718	72.08 ± 11.176	0.010
Heart rate (beats/min)	84.99 ± 15.12	88.10 ± 17.831	81.89 ± 12.422	0.034
Respiratory (rate /min)	16.73 ± 1.44	17.57 ± 1.484	15.89 ± 1.413	< 0.0001
Temperature (°C)	36.88 ± 0.2172	36.819 ± 0.2391	36.942 ± 0.1954	0.002
Systolic BP (mmHg)	111.67 ± 7.61	108.55 ± 7.120	114.80 ± 8.112	< 0.0001
Diastolic BP (mmHg)	68.64 ± 6.91	68.36 ± 6.351	68.93 ± 7.486	0.63
Gestational age (days)	269.50 ± 11.51	271.40 ± 11.050	267.61 ± 11.974	0.052
Hemoglobin before delivery (g/dL)	11.69 ± 1.45	10.995 ± 1.7813	12.387 ± 1.1253	< 0.0001
Hemoglobin after delivery (g/dL)	10.02 ± 1.36	8.840 ± 1.5717	11.210 ± 1.1661	< 0.0001
Neonatal weight (g)	3095.85 ± 495.215	3080.36 ± 506.959	3111.48 ± 411.472	0.654
Gravida	2.38 ± 1.25	2.40 ± 1.432	2.36 ± 1.068	0.210

Data are presented as mean ± standard deviation. An independent t-test or Mann–Whitney U test was used to compare groups according to the results of the normality test

A p-value < 0.05 was considered statistically significant

All cases had normal blood loss, Perineal Status and Suture Site, Uterine Status and urinary output. All cesarean deliveries (*n* = 305) received spinal anesthesia, and all transfused patients (*n* = 42) received packed cells, which precluded comparative statistical analysis for these variables.

When all deliveries were analyzed together, a chi-square test showed a significant association between categorized SI and the need for blood transfusion (*P* < 0.001). However, this association was not statistically significant when analyses were stratified by delivery type. Among 370 women studied, 42 (11.4%) required blood transfusion. The highest proportion of transfusions was observed in the SI 0.7–0.89 group (40.5%), followed by the SI ≥ 0.9 group (28.6%) and the SI < 0.7 group (31.0%), indicating that higher SI values, particularly in the 0.7–0.89 range, were more common in transfused women. Additionally, vital signs were compared between the transfusion (*n* = 42) and non-transfusion (*n* = 328) groups using the Mann-Whitney U test. Women requiring transfusion had a higher mean heart rate (88.1 ± 17.8 vs. 81.9 ± 12.4 beats per minute) and SI (0.81 ± 0.18 vs. 0.72 ± 0.11), but lower mean systolic blood pressure (108.6 ± 7.1 vs. 114.8 ± 8.1 mmHg), supporting the association between higher SI values and the need for transfusion post‑delivery.

Table 3 summarizes the two-way ANOVA results assessing the effects of categorized SI and episiotomy status on pre-delivery hemoglobin levels in vaginal deliveries, stratified by transfusion status. Among women who required transfusion, categorized SI explained 45.7% of the variance in pre-delivery hemoglobin levels, while in those who did not require transfusion it accounted for 12.2% of the variance; however, no statistically significant differences were observed across SI categories or episiotomy status in either group (*P* > 0.05) (Table 3).

**Table 3:** Effect of SI and episiotomy status on pre delivery hemoglobin and transfusion requirements in vaginal deliveries

Category	Variable	Mean Square	F	*P*-Value
Transfusion(+)	Intercept	2267.081	1634.091	- 0.348 0.836
	categorized SI	1.578	1.138	
	Episiotomy	0.062	0.045	
=0.457, .adj = 0.263				
Transfusion(-)	Intercept	1606.063	998.303	- 0.365 0.096
	categorized SI	1.662	1.033	
	Episiotomy	4.685	2.912	
=0.122, .adj = 0.034				

Table 4 summarizes the two-way ANOVA results assessing the effects of categorized SI and episiotomy status on post‑delivery hemoglobin levels in vaginal deliveries, stratified by transfusion status. Categorized SI explained 23.3% of the variance among women who required transfusion and 10.9% among those who did not; however, no statistically significant differences were observed across SI categories or episiotomy status in either group (*P* > 0.05) (Table 4).

**Table 4:** Effect of SI and episiotomy status on post delivery hemoglobin and transfusion requirements in vaginal deliveries

Category	Variable	Mean Square	F	*P*-Value
Transfusion(+)	Intercept	243.1409	649.677	- 0.410 0.974
	categorized SI	2.064	0.951	
	Episiotomy	0.002	0.001	
=0.233, .adj=-0.041				
Transfusion(-)	Intercept	1262.778	562.803	- 0.954 0.053
	categorized SI	0.105	0.047	
	Episiotomy	8.912	3.972	
=0.109, .adj = 0.020				

Table 5 summarizes the one-way ANOVA results evaluating the effect of categorized SI on pre‑delivery hemoglobin levels in cesarean deliveries, stratified by transfusion status. Categorized SI explained 13.6% of the variance among women who required transfusion and 0.3% among those who did not; however, no statistically significant differences were observed across categorized SI levels in either group (*P* > 0.05) (Table 5).

**Table 5:** Effect of SI on pre delivery hemoglobin and transfusion requirements in cesarean deliveries

Category	Variable	Mean Square	F	*P*-Value
Transfusion(+)	Intercept	2323.996	569.969	- 0.250
	categorized SI	6.094	1.495	
=0.136, .adj = 0.045				
Transfusion(-)	Intercept	18490.839	15269.218	- 0.673
	categorized SI	0.479	0.396	
=0.003, .adj=-0.004				

Table 6 summarizes the one-way ANOVA results evaluating the effect of categorized SI on post‑delivery hemoglobin levels in cesarean deliveries, stratified by transfusion status. Categorized SI explained 8.5% of the variance among women who required transfusion and 1.2% among those who did not; however, no statistically significant differences were observed across categorized SI levels in either group (*P* > 0.05) (Table 6).

**Table 6:** Effect of SI on post delivery hemoglobin and transfusion requirements in cesaren deliveries

Category	Variable	Mean Square	F	*P*-Value
Transfusion(+)	Intercept	1603.477	540.459	- 0.430
	categorized SI	2.621	0.883	
=0.085, .adj=-0.011				
Transfusion(-)	Intercept	15029.784	12436.327	- 0.176
	categorized SI	2.115	1.750	
=0.012, .adj = 0.005				

## Discussion

In this retrospective study, systolic blood pressure and heart rate recorded during the fourth 15-minute interval post-delivery were extracted from medical records to calculate the SI.

Among 370 postpartum women, only 42 (11.4%) required blood transfusion. This relatively high transfusion rate may be partly explained by the high proportion of cesarean deliveries in our study population (82.4%). Global and regional evidence indicates that cesarean section rates have increased substantially over recent decades and exceed the World Health Organization recommended threshold in several countries, including Iran, particularly in referral and academic hospital settings [16].

The findings of this study indicate that the SI was not significantly associated with postpartum hemoglobin decline or transfusion requirement when analyses were stratified by delivery type. However, simple group comparisons between women who required transfusion and those who did not showed significantly higher heart rates, lower systolic blood pressure, and higher SI values in the transfusion group (*P* < 0.05). These differences suggest early hemodynamic variation among women who eventually required transfusion, although no clear association with hemoglobin decline within delivery subgroups was demonstrated.

Importantly, this study did not include formal predictive modeling such as regression‑based prediction analysis, ROC curve analysis, or sensitivity and specificity estimation. Therefore, the observed differences between groups should be interpreted as associations rather than predictive performance, and no diagnostic accuracy or screening capability can be inferred from these results. Incomplete documentation of vital signs in medical records may also have contributed to the non‑significant findings in stratified analyses.

Future prospective studies with standardized data collection, larger sample sizes, and formal predictive performance assessment are needed to more rigorously evaluate whether the SI can serve as a clinically useful tool for early identification of postpartum complications.

Postpartum hemorrhage (PPH), alongside hypertensive disorders and infection, is a leading preventable cause of maternal mortality, with nearly one-third of severe cases preventable [17]. Early identification and standard management of postpartum hemorrhage, supported by recent evidence, enhance maternal survival by improving hemodynamic stability and also benefit neonatal outcomes [18, 19]. Pregnant women may tolerate up to 1000 mL of blood loss without overt hypovolemic symptoms, with tachycardia, tachypnea, and hypotension typically manifesting after a loss of at least 1500 mL (over 25% of total blood volume). Consequently, the delayed clinical presentation of hypovolemia often indicates advanced bleeding, making rapid diagnosis of postpartum hemorrhage challenging [9, 10]. The SI, which normally ranges from 0.5 to 0.7 in non-pregnant individuals and 0.7 to 0.9 during pregnancy, serves as a sensitive indicator of compensated hypovolemia [5]. Unlike visual blood loss estimation, which underestimates volume by up to 50%, SI provides a reliable early warning of critical deterioration [20].

Castillo-Reyther et al. [21] reported a significant difference in the SI between the severe and non-severe hemorrhage groups both at the time of diagnosis and 30 min following diagnosis (0.7 ± 0.2 versus 0.9 ± 0.38). Capillary lactate level 30 min post-diagnosis effectively discriminated the severe group from the non-severe group, whereas capillary hemoglobin was unable to differentiate between the two groups during this interval [21]. Consistently, both studies demonstrated that there is no significant association between hemoglobin and severe hemorrhage in the early stages of bleeding onset. This finding affirms the limited role of hemoglobin as an early indicator for hemorrhage identification. This observation is attributed to the delayed nature of the drop in hemoglobin during acute hemorrhage, as its changes are influenced by blood volume and fluid shifts and are typically observed in later stages of bleeding [6].

Our findings are partially consistent with those of Drew et al. (2021), who demonstrated that the SI significantly correlates with postpartum hemorrhage within 30 min post-delivery, while hemoglobin levels exhibit a delayed decline, limiting their utility as an early marker. The lack of direct correlation between SI and hemoglobin in our study may reflect their differing temporal responses, with hemoglobin changes lagging due to hemodilution from fluid administration [22]. These results suggest that the SI may reflect early hemodynamic changes before measurable declines in hemoglobin occur. However, because we did not assess diagnostic accuracy (e.g., sensitivity, specificity, ROC/AUC, or optimal cut‑off values), our findings cannot establish the SI as a screening test and instead indicate only an association between higher SI values and subsequent transfusion.

Mizutani et al. (2024) found that the SI exhibits high predictive accuracy for postpartum hemorrhage in vaginal deliveries but low accuracy in cesarean Sect [23]. This inconsistency with our findings may be attributed to differences in sample sizes, which likely influenced the statistical power needed to detect a consistent association between the SI and postpartum hemorrhage.

A case-control study by Borovac-Pinheiro et al. (2018) found that although SI was higher in the transfusion group, it did not reach statistical significance in the cesarean subgroup (*P* > 0.05). This finding aligns with our results, where SI failed to predict hemoglobin decline or transfusion requirement in cesarean deliveries [24]. The authors suggested that in cesarean sections, the rapid hemodynamic changes associated with anesthesia and surgical stress may obscure the isolated effect of hemorrhage on SI, limiting its discriminative power [23]. Consistent with our findings, Madar et al. (2024) reported that the SI measured 15 min postpartum is a poor predictor of PPH in women with vaginal deliveries. Nevertheless, its high Negative Predictive Value (NPV) may guide the duration of postpartum surveillance, particularly in settings with limited resources. To validate this utility, further interventional studies are required [25].

The present study has several strengths. It evaluated the SI as a simple, non-invasive, and readily available bedside parameter derived from routine vital signs, making it particularly applicable in resource-limited clinical settings. The SI was measured during a critical early postpartum period (the fourth 15-minute interval after delivery), when timely recognition of hemodynamic instability is essential. In addition, the study was conducted across three public tertiary referral hospitals, enhancing the clinical relevance of the findings to similar high-volume and referral-level settings. The use of clinically meaningful SI categories and stratified analyses by mode of delivery and episiotomy status further supports the interpretability of the results and allows a more nuanced assessment across different obstetric contexts.

The limitations of the present study include its retrospective design, a relatively small sample size, and measurement at a single time interval (the fourth 15 min postpartum), and reliance on medical records with potential for bias. Additionally, the exclusion of women under 18 years of age limits the generalizability of the results to adolescent pregnancies.

Another limitation of this study is that the relationship between SI and the number of transfused blood units could not be evaluated, as this variable was not included in the initial data collection checklist and therefore was not collected for analysis.

Future prospective, multicenter studies incorporating serial SI measurements and larger sample sizes are warranted to enable more standardized data collection, more accurate quantification of blood loss, and a clearer assessment of the clinical utility of SI.

## Conclusion

Our findings indicate that women who required blood transfusion, regardless of delivery mode, exhibited significantly higher mean heart rates, lower systolic blood pressure, and higher SI values compared with those who did not. Although these group differences were statistically significant, subgroup analyses by delivery type (vaginal delivery with or without episiotomy and cesarean section) showed no significant association between categorized SI and postpartum hemoglobin levels or transfusion requirement. These findings may partly reflect the relatively small number of transfusion cases and incomplete documentation of certain clinical parameters.Because this study examined only associations and did not include diagnostic accuracy analyses such as sensitivity, specificity, ROC/AUC, or determination of optimal SI cutoff values, the SI cannot be considered a screening tool based on our results. Future prospective studies with larger sample sizes and standardized or automated vital sign monitoring are needed to more accurately assess the potential diagnostic or screening utility of the SI and determine whether it could aid in the early identification of mothers at risk of postpartum hemorrhage.

## Supplementary Information

Supplementary Material 1.

## Data Availability

The datasets analyzed during this study are not publicly available due to privacy and ethical restrictions but may be available from the corresponding author upon reasonable request and with approval from the Ethics Committee of Shiraz University of Medical Sciences.
