# Supplementary material for: Association between shock index and postpartum hemoglobin level and blood transfusion requirement in women delivering at public hospitals in Shiraz, 2023–2024
Source: BMC Pregnancy Childbirth. 2026 May 29;26:828. doi: 10.1186/s12884-026-09323-7 (PMC13420852; doi:10.1186/s12884-026-09323-7)
Supplement: Supplementary file 1 — Supplementary Material 1. [file 12884_2026_9323_MOESM1_ESM.docx]

**Obstetric Data Checklist**

|  | **Obstetric Data Checklist** |  |
| --- | --- | --- |
| 1 | Maternal Age |  |
| 2 | Height |  |
| 3 | Weight |  |
| 4 | Pulse Rate |  |
| 5 | Respiratory Rate |  |
| 6 | Temperature |  |
| 7 | Systolic Blood Pressure |  |
| 8 | Systolic Blood Pressure |  |
| 9 | Uterine Status |  |
| 10 | Perineal and Suture Status |  |
| 11 | Blood Loss |  |
| 12 | Gestational Age |  |
| 13 | Pre-delivery Hemoglobin Level |  |
| 14 | Post-delivery Hemoglobin Level |  |
| 15 | Blood Transfusion at the end of the first hour |  |
| 16 | Urine Output for Cesarean Section and Vaginal |  |
| 17 | Newborn Weight |  |
| 18 | Episiotomy Status |  |
| 19 | Episiotomy Grade |  |
| 20 | Gravida |  |
| 21 | Parity |  |
| 22 | Number of Abortions |  |
| 23 | Delivery Method |  |
| 24 | Instrumental Delivery |  |
| 25 | Medications Used |  |
| 26 | Type of Blood Product |  |
| 27 | Type of Anesthesia |  |
| 28 | Neonatal status |  |
